# Supplementary material for: Evidence for intrinsic charm quarks in the proton
Source: Nature. 2022 Aug 17;608(7923):483–7. doi: 10.1038/s41586-022-04998-2 (PMC9385499; doi:10.1038/s41586-022-04998-2)
Supplement: Supplementary file 1 — In Section A we summarize the main features of the EKO evolution framework used to transform the charm PDF from the 4FNS to the 3FNS. In Section B we discuss the perturbative charm PDF in the light of the N3LO matching conditions and its dependence on the charm quark mass. In Section C and Section D, respectively, we assess the stability of the charm PDF in the 4FNS and 3FNS with respect to a number of data and methodological variations. In Section E, we study the implications of our determination of the charm PDF for the charm momentum fraction in the proton, and in Section F we compare our results with those based on the CT14IC global analysis of intrinsic charm. In Section G, we present technical details related to the calculation of the Z + charm production in the forward region, and finally we compare partonic luminosities in the PDF determinations with and without intrinsic charm among them for the kinematics of central and forward charm production at the LHC. [file 41586_2022_4998_MOESM1_ESM.pdf]

nature portfolio

https://doi.org/10.1038/s41586-022-04998-2

Supplementary information

Evidence for intrinsic charm quarks in the proton

In the format provided by the authors and unedited

Edinburgh 2021/28
Nikhef 2021-032
TIF-UNIMI-2021-21

Evidence for intrinsic charm quarks in the proton

The NNPDF Collaboration:

Richard D. Ball,[1] Alessandro Candido,[2] Juan Cruz-Martinez,[2] Stefano Forte,[2] Tommaso Giani,[3,4] Felix Hekhorn,[2] Kirill Kudashkin,[2] Giacomo Magni,[3,4] and Juan Rojo[3,4†]

[1] *The Higgs Centre for Theoretical Physics, University of Edinburgh, JCMB, KB, Mayfield Rd, Edinburgh EH9 3JZ, Scotland*

[2] *Tif Lab, Dipartimento di Fisica, Università di Milano and INFN, Sezione di Milano, Via Celoria 16, I-20133 Milano, Italy*

[3] *Department of Physics and Astronomy, Vrije Universiteit, NL-1081 HV Amsterdam*

[4] *Nikhef Theory Group, Science Park 105, 1098 XG Amsterdam, The Netherlands*

Supplementary Information

In this Supplementary Information, references that already appear in the main manuscript use the same number, and new references are provided at the end of the document.

Contents

A The EKO evolution framework 2

B The perturbative charm PDF 4

C Stability of the 4FNS charm PDF 5

D Stability of the 3FNS charm calculation 9

E The charm momentum fraction 12

F Comparison with CT14IC 15

G Z+charm production in the forward region 17

H Parton luminosities 19

†Corresponding author (j.rojo@vu.nl).

A The EKO evolution framework

A crucial ingredient in the derivation of our results is the determination of the 3FNS intrinsic charm PDF starting from the 4FNS, which requires the inversion of the matching conditions implementing the 3FNS to the 4FNS transformation. This inversion is not available in the open-source NNPDF code [51] and it is performed here by means of a novel code for QCD evolution, EKO (Evolution Kernel Operators), that we use to take the PDF set determined at the reference scale Q_0 and evolve it to a matching scale Q_c where it is transformed to the 3FNS. Here we provide a brief summary of EKO, some details of the way the direct and inverse matching conditions are implemented, and some benchmarks between EKO and other existing QCD evolution codes, including the APFEL [52] evolution code that is used by the NNPDF code. EKO is written in PYTHON and is available open source from its GITHUB repository

https://github.com/N3PDF/eko

A more detailed description of the code can be found in its online documentation

https://eko.readthedocs.io/en/latest/

as well as in a dedicated publication [53].

The scale dependence of PDFs in QCD is determined by solving a set of coupled integro-differential equations (evolution equations) in two variables x (momentum fraction) and Q (scale) on which PDFs depend. Two families of approaches are commonly used in order to this purpose. One possibility is to treat the (integral) dependence on x of the PDFs and evolution kernels by sampling it on a grid of points. This is the strategy adopted by, among others, the APFEL [52], HOPPET [54], and QCDNUM [55] evolution codes. An alternative possibility is to perform an integral transform (Mellin transform) with respect to x thereby turning the integro-differential equations into coupled ordinary differential equations. These can then be solved analytically, but the integral transform has to be inverted numerically to arrive at a final result. This approach is adopted by PEGASUS [56] as well as by the internal PDF evolution code FASTKERNEL used in earlier NNPDF analyses and described in [57–59]. One limitation of PEGASUS is that it requires the analytic computation of the Mellin transforms of the PDFs, which is generally not possible, specifically if PDFs are parametrized as neural networks.

This restriction is bypassed in FASTKERNEL by transforming only the evolution kernel (i.e. the evolution operator, solution of differential equations, evaluated on a given interpolation basis), which can be then convoluted with the x-space PDFs at the input evolution scale Q_0. Following a similar strategy, EKO solves evolution equations in Mellin space and then produces PDF-independent evolution kernel operators (EKO) which can be convoluted with input PDFs. Variable flavor number scheme evolution (VFNS) is implemented in EKO, with the possibility of freely choosing the value of the matching scales Q_h between the N-flavor number scheme (NFNS) in which heavy quark h is not included in QCD evolution, and the $(N+1)$-flavor number scheme in which it is included. Schematically, for evolution between Q_0 and Q_1 if no matching scales are crossed $\left(Q_h^2 < Q_0^2, Q_1^2 < Q_{h'}^2\right)$ one has:

$$\mathbf{f}^{(n_f)}(Q_1^2) = \mathbf{E}^{(n_f)}(Q_1^2 \leftarrow Q_0^2) \otimes \mathbf{f}^{(n_f)}(Q_0^2)\,, \tag{A.1}$$

where $\mathbf{E}^{(n_f)}$ is the NFNS EKO, and $\otimes$ is the Mellin convolution operation. Note that EKO can perform both "forward" ($Q_0 < Q_1$) and "backward" ($Q_1 < Q_0$) evolution. Bold quantities indicate either vectors or matrices in the $(2n_f + 1)$-dimensional flavor space. If instead a single matching scale Q_h is crossed, assuming for definiteness $Q_0 < Q_1$, $Q_{h'}^2 < Q_0^2 < Q_h^2 < Q_1^2 < Q_{h''}^2$, one has

$$\mathbf{f}^{(n_f+1)}(Q_1^2) = \left[\mathbf{E}^{(n_f+1)}(Q_1^2 \leftarrow Q_h^2)\mathbf{A}^{(n_f)}(Q_h^2)\mathbf{E}^{(n_f)}(Q_h^2 \leftarrow Q_0^2)\right] \otimes \mathbf{f}^{(n_f)}(Q_0^2)\,, \tag{A.2}$$

where $\mathbf{A}^{(n_f)}(Q_h^2)$ is the scheme transformation between the NFNS and (N+1)FNS, given as a perturbatively computable series expansion in α_s. The quantity in square parenthesis is evaluated in Mellin space and then transformed to x-space. This procedure can be extended to the case in which more than one threshold is crossed. Also, the scales Q_0 and Q_1 can be

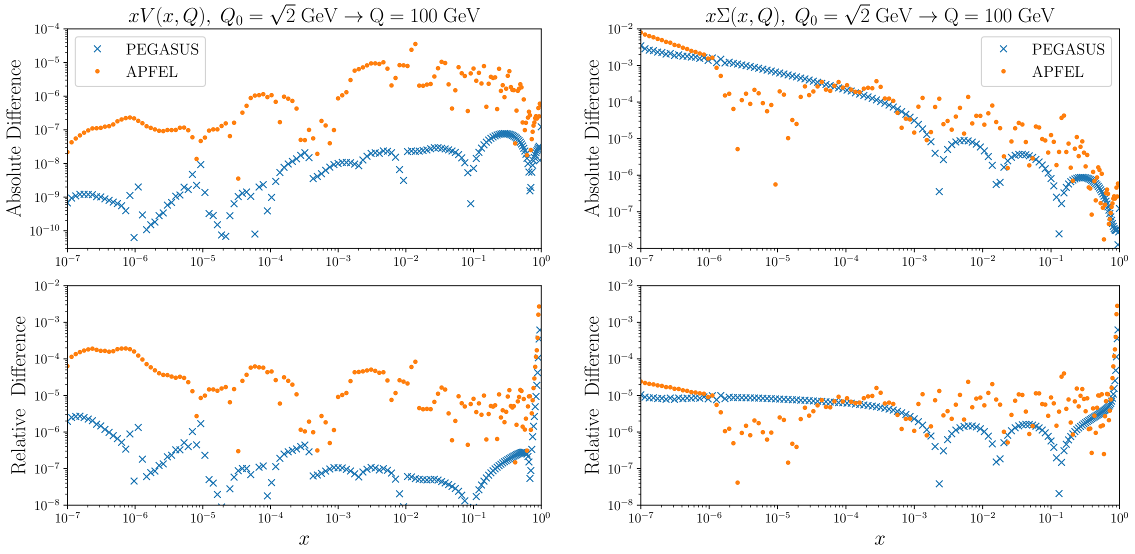

Figure A.1. Absolute (upper) and relative (bottom) differences between the outcome of NNLO QCD evolution as implemented in EKO and the corresponding results from APFEL and PEGASUS. We adopt the settings of the Les Houches PDF evolution benchmarks: we consider VFNS evolution from $Q_0 = \sqrt{2}$ GeV up to $Q = 100$ GeV, and we show results for the total valence quark distribution V (left) and the quark singlet distribution Σ (right).

ordered in any way, because both direct and inverse scheme transformations are implemented in EKO. Furthermore, the inverse scheme change is implemented both perturbatively (i.e. as a series expansion in α_s to the same accuracy as the direct scheme change) or exactly (i.e. as the numerical inverse, completely equivalent to the analytic one within the numeral accuracy of the rest of the calculation).

If the heavy quark h has no intrinsic component, then below Q_h its PDF is identically zero, and above Q_h it is determined by $\mathbf{A}^{(n_f)}(Q_h^2)$. If it does have an intrinsic component, then below Q_h its PDF is scale-independent, but nonzero. While EKO is currently an NNLO code, on top of the standard [25] NNLO scheme change for this work an N^{3}LO scheme change has been implemented, based on recent higher-order computations of the relevant operator matrix elements [26–34] and the work of [60].

The EKO implementation of QCD evolution has been benchmarked against the Les Houches PDF evolution benchmarks [61, 62] and with APFEL and PEGASUS, finding excellent agreement beyond the per-mille level. The implementation of the matching conditions has been benchmarked up to N^{3}LO against the independent MATHEMATICA-based calculation presented in [60] finding also good agreement. To illustrate some of these benchmarks, Fig. A.1 displays the absolute and relative difference between EKO, APFEL, and PEGASUS for NNLO VFNS evolution carried out following the settings of [61, 62]. A toy PDF set at $Q_0 = \sqrt{2}$ GeV is evolved up to $Q = 100$ GeV for equal values of the factorization and renormalization scales, $Q_f = Q_r = Q$. We show as representative results those corresponding to the total valence quark V and the quark singlet Σ distributions. Excellent agreement is found, in particular with PEGASUS which also perform QCD evolution in Mellin space, with relatively differences at most at the $\mathcal{O}\left(10^{-4}\right)$ level. A similar level of agreement is found for the gluon and for the other quark PDF combinations.

B The perturbative charm PDF

In the absence of intrinsic charm, the charm PDF is fully determined by perturbative matching conditions, i.e. by the matrix $\mathbf{A}^{(n_f)}(Q_c^2)$ in Eq. (A.2). We will denote the charm PDF thus obtained "perturbative charm PDF", for short. The PDF uncertainty on the perturbative charm PDF is directly related to that of the light quarks and especially the gluon, and is typically much smaller than the uncertainty on our default charm PDF, that includes intrinsic charm. Here and in the following we will refer to our final result, as shown in Fig. 1 (right) as "default". It should be noticed that the matching conditions for charm are nontrivial starting at NNLO: at NLO the perturbative charm PDF vanishes at threshold. Hence, having implemented in EKO also the N^{3}LO matching conditions, we are able to assess the MHOU of the perturbative charm at the matching scale Q_c, by comparing results obtained at the first two nonvanishing perturbative orders.

As already mentioned, see also Fig. 2 (top left) in the main manuscript, we have constructed a PDF set with perturbative charm, in which the full PDF determination from the global dataset leading to the NNPDF4.0 PDF set is repeated, but now with the assumption of vanishing intrinsic charm, i.e. with a perturbative charm PDF. This perturbative charm PDF is compared to our default result in Fig. B.1 (left), where the 4FNS perturbative charm PDF at scale $Q_c = m_c$ obtained using either NNLO or N^{3}LO under the assumption of no intrinsic charm are shown, together with our result allowing for intrinsic charm. It is clear that while on the one hand, the PDF uncertainty on the perturbative charm PDF is indeed tiny, on the other hand the difference between the result for perturbative charm obtained using NNLO or N^{3}LO matching is large, and in fact larger at small x than the difference between perturbative charm and our default (intrinsic) result.

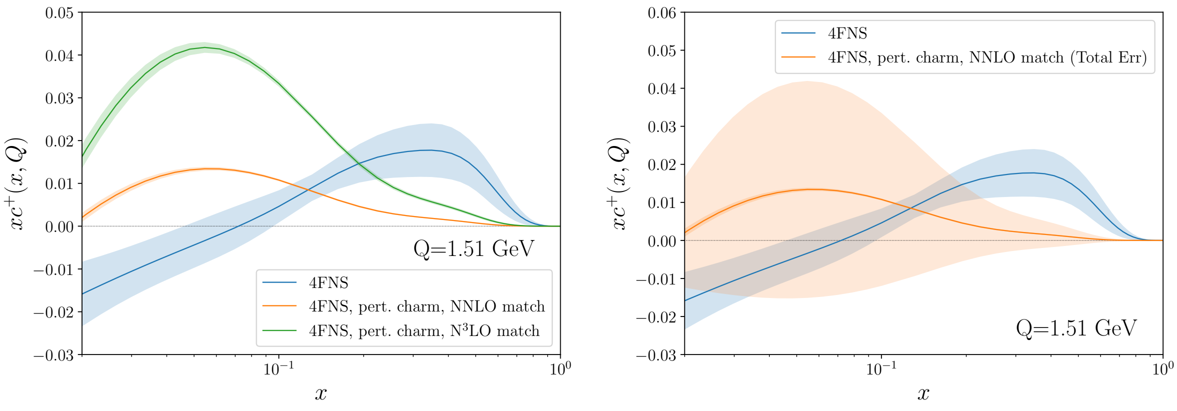

Figure B.1. Left: the perturbative charm PDF at $Q = 1.51$ GeV obtained from NNLO PDFs using NNLO and N^{3}LO matching conditions. Right: the NNLO perturbative charm PDF including the MHOU computed as the difference between NNLO and N^{3}LO matching. In both plots our default (intrinsic) charm PDF is also shown for comparison.

In the same manner as we used the difference between the results obtained from inversion of NNLO and N^{3}LO matching as an estimate of the MHOU on intrinsic charm, we may use the difference between the 4FNS perturbative charm obtained from NNLO and N^{3}LO matching as an estimate of the MHOU on perturbative charm at the scale Q_c. The total uncertainty is found by adding this in quadrature to the PDF uncertainty (which however in practice is negligible). The result is shown in Fig. B.1 (right). Within this total uncertainty there is now good agreement between our intrinsic charm result and perturbative charm for all $x \lesssim 0.2$. On the other hand, there is a clear deviation for larger x. We may view the difference between the 4FNS default result and the 4FNS perturbative charm as the intrinsic component in the 4FNS, and indeed it is clear from Fig. B.1 that the 4FNS intrinsic component is sizable and positive at large x. This is of course consistent with our main finding that we only see evidence of intrinsic charm for large $x \gtrsim 0.2$, while for smaller x our result for the charm PDF is compatible with zero, as demonstrated by Fig. 1 (right) in the main manuscript.

C Stability of the 4FNS charm PDF

The main input to our determination of intrinsic charm is the 4FNS charm PDF extracted from high-energy data. While this determination comes with an uncertainty estimate, it is important to verify that this adequately reflects the various sources of uncertainty, and that there are no further sources of uncertainty that may be unaccounted for. To this purpose, here we assess the stability of our results first, upon the choice of underlying dataset, next upon changes in methodology, and finally, upon variation of standard model parameters. In each case we verify stability upon the most important possible source of instability: respectively, the use of collider vs. fixed target and deep-inelastic vs. hadronic data (dataset); the choice of parametrization basis (methodology); and the value of the charm quark mass (standard model parameters). As a final consistency check, we compare our result with that which we would have obtained by using the same input dataset, but the previous NNPDF3.1 fitting methodology. Because we are interested in intrinsic charm, in all comparisons we focus on the large-x region in which the intrinsic valence-like peak is found. In this section, the 4FNS charm PDF is displayed at the scale $Q = 1.65$ GeV so that results for all fit variants, including those with with different m_c values, can be shown at a common scale.

Dependence on the choice of dataset. We now study the stability of the 4FNS charm determination upon variation of the underlying data, which also allows us to identify the datasets or groups of processes that provide the leading constraints on intrinsic charm. To this purpose, we have repeated our PDF determination using a variety of subsets of the global dataset used for our default determination. Results are shown in Fig. C.1, where we compare the result using the baseline dataset to determinations performed by adding to the baseline the EMC charm structure function data (already discussed in the main text); by only including DIS data; by only including collider data (HERA, Tevatron and LHC); and by removing the LHCb W and Z production data.

As already noted in the main text in the case of the 3FNS result, we find that the extra information provided by the EMC F_2^c data is subdominant in comparison to that from the global dataset. The result is stable and only a moderate uncertainty reduction at the peak is observed. It is interesting to contrast this with the previous NNPDF study [22], in which the global fit provided only very loose constraints on the charm PDF, which was then determined mostly by the EMC data. Indeed, a DIS-only fit (for which most data were already available at the time of the previous determination) determines charm with very large uncertainties. On the other hand, both the central value and uncertainty found in the collider-only fit are quite similar to the baseline result. This shows that the dominant constraint is now coming from collider, and specifically hadron collider data (indeed, as we have seen constraints from DIS data are quite loose). Among these, LHCb data (which are taken at large rapidity and thus impact PDFs at large and small x) are especially important, as demonstrated by the increase in uncertainty when they are removed.

In all these determinations, the charm PDF at $x \simeq 0.4$ remains consistently nonzero and positive, thus emphasizing the stability of our results.

Dependence on the parametrization basis. Among the various methodological choices, a possibly critical one is the choice of basis functions. Specifically, in our default analysis, the output of the neural network does not provide the individual quark flavor and antiflavor PDFs, but rather linear combinations corresponding to the so-called evolution basis [3]. Indeed, our charm PDF is given in Eq. (1) as the linear combination of the two basis PDFs Σ and T_{15}. One may thus ask whether this choice may influence the final results for individual quark flavors, specifically charm. Given that physical results are basis independent, the outcome of a PDF determination should not depend on the basis choice.

In order to check this, we have repeated the PDF determination, but now using the flavor basis, see Sect. 3.1 of [3], in which each of the neural network output neurons now correspond to individual quark flavors, so in particular, instead of Eq. (1), one has

$$xc^+(x, Q_0; \boldsymbol{\theta}) = (1 - x)^{\beta_{c^+}} \mathrm{NN}_{c^+}(x, \boldsymbol{\theta}) \,, \qquad \text{(C.1)}$$

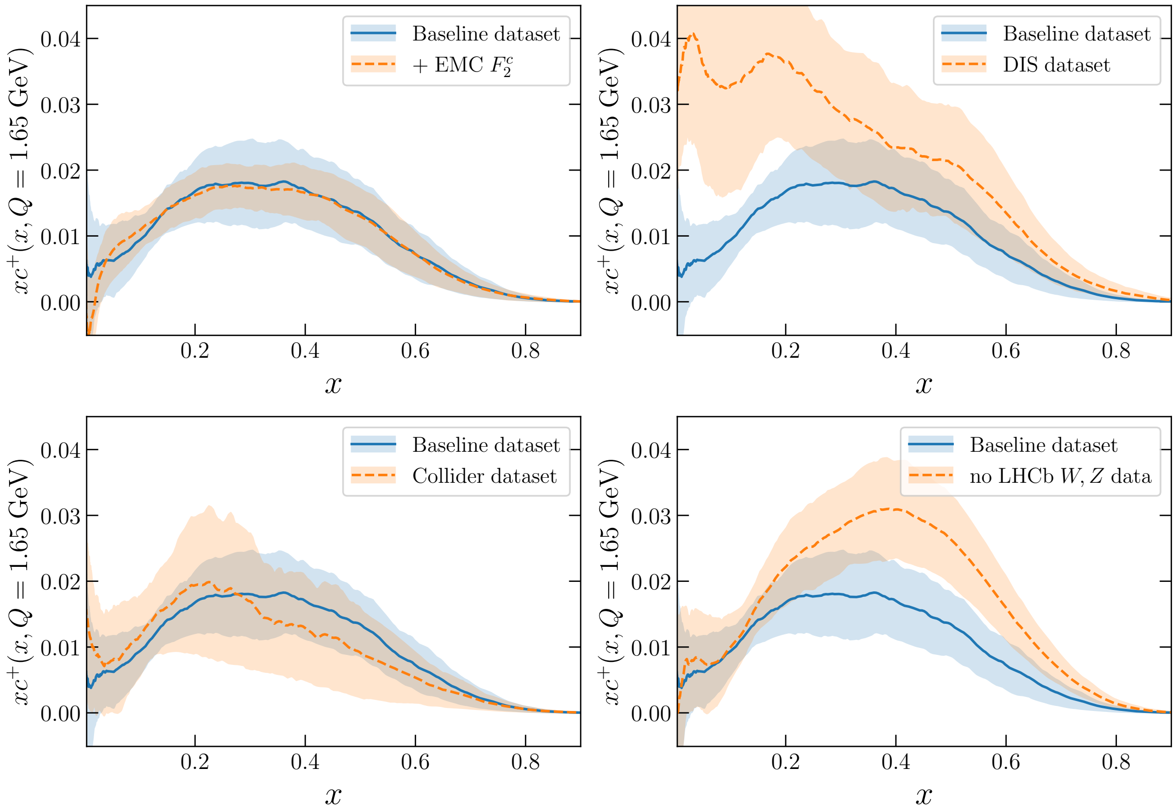

Figure C.1. The dependence of the 4FNS charm PDF at $Q = 1.65$ GeV on the input dataset. We compare the baseline result with that obtained by also including EMC F_2^c data (top left), only including DIS data (top right), only including collider data (bottom left) and removing LHCb gauge boson production data (bottom right).

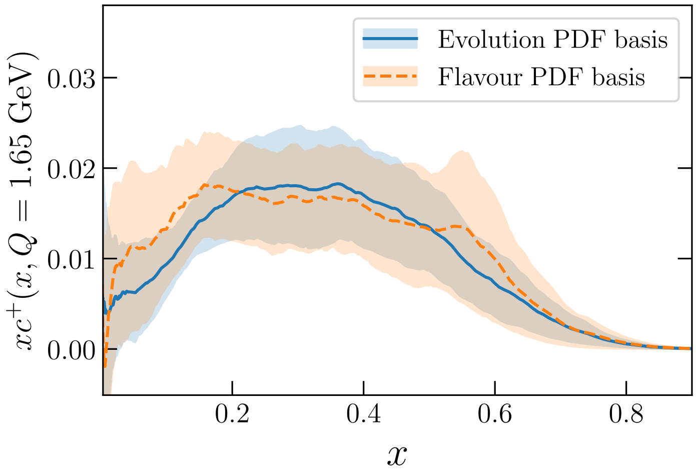

Figure C.2. The default 4FNS charm PDF at $Q = 1.65$ GeV compared to a result obtained by parametrizing PDFs in the flavor basis instead of the evolution basis.

where $\text{NN}_{c^+}(x, \boldsymbol{\theta})$ indicates the value of the output neuron associated to the charm PDF c^+. The 4FNS charm PDFs determined using either basis are compared in Fig. C.2 at $Q = 1.65$ GeV. We find excellent consistency, and in particular the valence-like structure at high-x is independent of the choice of parametrization basis.

Dependence on the charm mass. The kinematic threshold for producing charm perturbatively depends on the value of the charm mass. Therefore the perturbative contribution to the

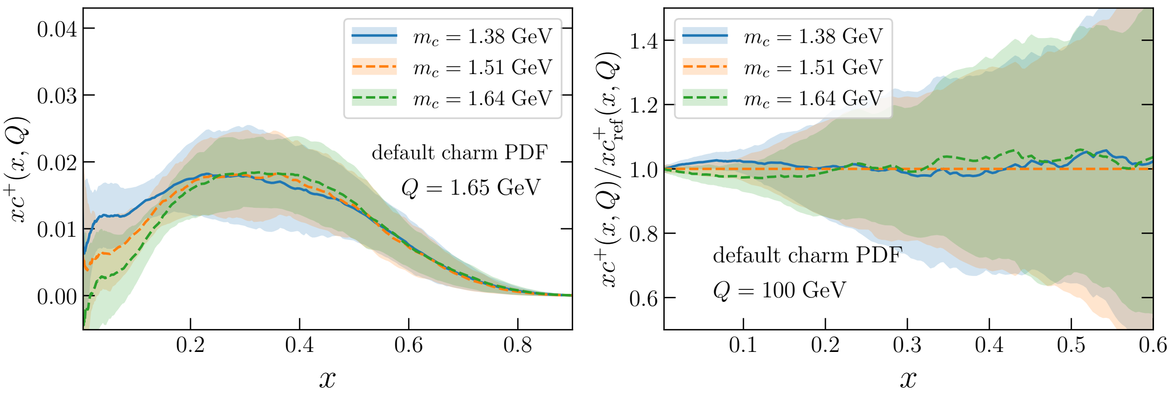

Figure C.3. The 4FNS charm PDF determined using three different values of the charm mass. The absolute result (left) is shown at $Q = 1.65$ GeV, while the ratio to the default value $m_c = 1.51$ GeV (right) used elsewhere in this paper is shown at $Q = 100$ GeV.

4FNS charm PDF, and thus the whole charm PDF if one assumes perturbative charm, depends strongly on the value of the charm mass. On the other hand, the intrinsic charm PDF is of nonperturbative origin, so it should be essentially independent of the numerical value of the charm mass that is used in perturbative computations employed in its determination (though it will of course depend on the true underlying physical value of the charm mass).

In order to study this mass dependence, we have repeated our determination using different values for the charm mass. The definition of the charm mass which is relevant for kinematic thresholds is the pole mass, for which we adopt the value recommended by the Higgs cross-section working group [63] based on the study of [64], namely $m_c = 1.51 \pm 0.13$ GeV. Results are shown in Fig. C.3, where our default charm PDF determination with $m_c = 1.51$ GeV is repeated with $m_c = 1.38$ GeV and $m_c = 1.64$ GeV. In order to understand these results note that this is the 4FNS PDF, so it includes both a nonperturbative and a perturbative component. The latter is strongly dependent on the charm mass, but of course the data correspond to the unique true value of the mass and the mass dependence of the perturbative component is present only due to our ignorance of the actual true value. When determining the PDF from the data, as we do, we expect this spurious dependence to be to some extent reabsorbed into the fitted PDF. So we expect results to display a moderate dependence on the charm mass — full independence should hold for the intrinsic (3FNS) PDF and will be investigated in SI Sect. D.

In Fig. C.4 the same result is shown, but now for the perturbative charm PDF discussed in SI Sect. B, so the charm PDF is of purely perturbative origin and fully determined by the strongly mass-dependent matching condition. This dependence is clearly seen in the plots. Furthermore, comparison with Fig. C.3 shows that indeed this spurious dependence is partly reabsorbed in the fit when the charm PDF is determined from the data, so that the residual mass dependence is moderate. In particular, the large-x valence peak, which is dominated by the intrinsic component, is very stable.

Comparison with NNPDF3.1. Fig. C.5 compares the baseline determination of the 4FNS charm PDF based on NNPDF4.0 with that obtained from the same input dataset but using instead the NNPDF3.1 fitting methodology and related settings such those related to positivity and integrability. Results are fully consistent between the two methodologies, with our default determination exhibiting reduced uncertainties due to the various improvements implemented in the NNPDF4.0 analysis framework.

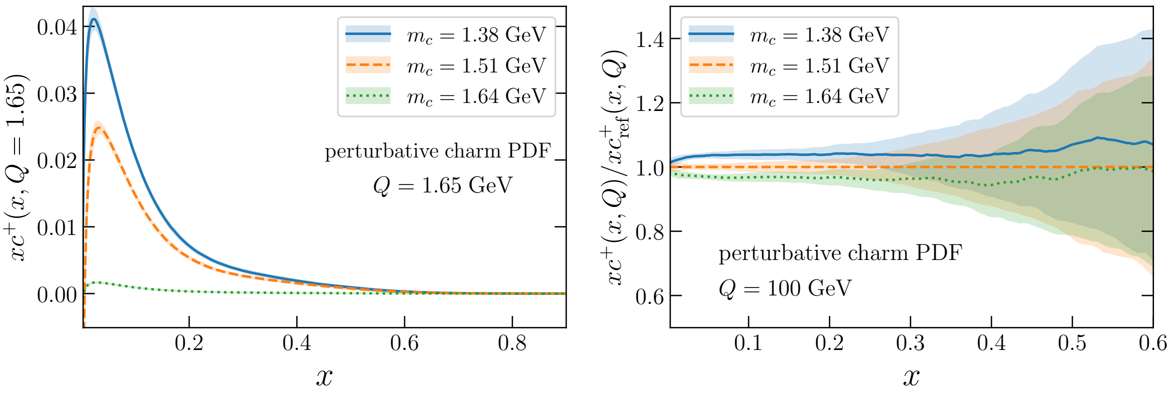

Figure C.4. The same as Fig. C.3 but now for the perturbative charm PDF.

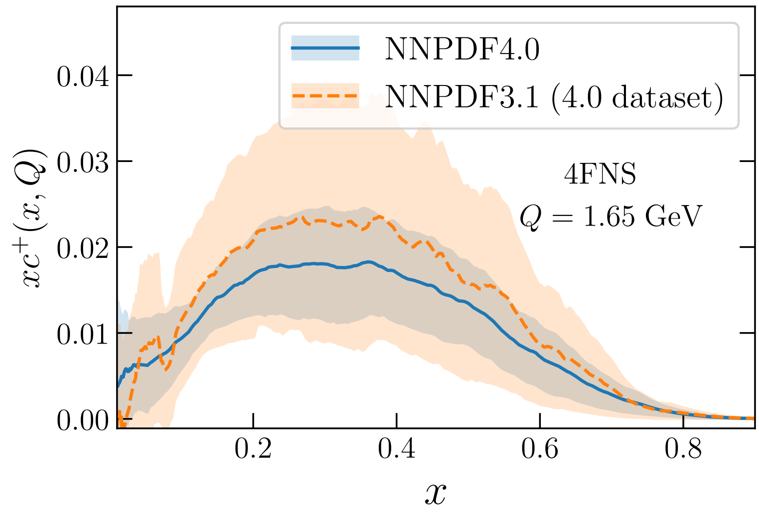

Figure C.5. Same as Fig. C.2, comparing the baseline determination of the 4FNS charm PDF, based on NNPDF4.0, with that obtained from the same dataset using the NNPDF3.1 fitting methodology.

D Stability of the 3FNS charm calculation

We now repeat the stability and uncertainty study of the previous section, but for our final result, namely the intrinsic charm PDF. The main difference to be kept in mind is that the uncertainty now also includes the dominant MHOU, due to the matching condition required in order to determine the 3FNS PDF from the 4FNS result. In order to get a complete picture, we now add a further set of dataset variations.

Dependence on the input dataset. Fig. D.1 displays the dataset variations shown in Fig. C.1, now for the intrinsic (3FNS) charm PDF, but with the total uncertainty now being the sum in quadrature of the PDF and MHO uncertainties, with the latter determined as the difference between results obtained using NNLO and N^{3}LO matching. Additionally, we also performed a few extra dataset variations: a fit without any W, Z production data from ATLAS and CMS, a fit without jet data, a fit without Z p_T measurements, and a fit without HERA structure function data. Note that the collider-only dataset includes both HERA electron-proton collider data and Tevatron and LHC hadron collider data, but not fixed-target deep-inelastic scattering and Drell-Yan production data.

Results are qualitatively very similar to those seen in the 4FNS, a consequence of the fact that we are focusing on the large-x region where the effect of the matching is moderate, though now the presence of a valence-like peak in all determinations is even clearer, specifically for the DIS-only fit where it was less pronounced in the 4FNS. Note however that the DIS-only determination exhibits larger uncertainties (up to factor 2) and point-by-point fluctuations, and is dominated by relatively old fixed-target measurements. Comparison of all the dataset variations shows that, in terms of their impact on intrinsic charm, hadron collider data are generally more important that deep-inelastic data, that among the former the LHCb inclusive W, Z data are playing a dominant role, and that jet observables also play a non-negligible role.

It should be stressed that the agreement between results found using DIS data and hadron collider data is highly nontrivial, since in the region relevant for intrinsic charm these determinations are based on disjoint datasets and are affected by very different theoretical and experimental uncertainties: in particular, potential higher-twist effects in the DIS observables are highly suppressed for collider observables. this respect, a DIS-only determination of intrinsic charm is potentially affected by sources of theory uncertainties, such as higher twists, which are not accounted for in global PDF determinations.

We conclude that the characteristic valence-like peak structure at large-x predicted by non-perturbative intrinsic charm models (Fig. 1 in the main manuscript) is always present even under very significant changes of the dataset.

Dependence on the parametrization basis. Fig. D.2 displays the comparison between the intrinsic charm PDF determined with the default evolution basis choice, and the flavor basis. Complete consistency of central values is found, with somewhat larger uncertainties in the case of the flavor basis, due to the more challenging fitting environment in this basis (see the discussion in [3]).

Dependence on the charm mass value. The study of the charm mass dependence is particularly interesting, because the intrinsic component should be independent of it, hence the residual dependence seen in Fig. C.3 in the 4FNS, due to the mass dependence of the perturbative component that could not be reabsorbed in the fitting, should no longer be present. Results are shown in Fig. D.3, and it is apparent that indeed the dependence on the charm mass has all but disappeared.

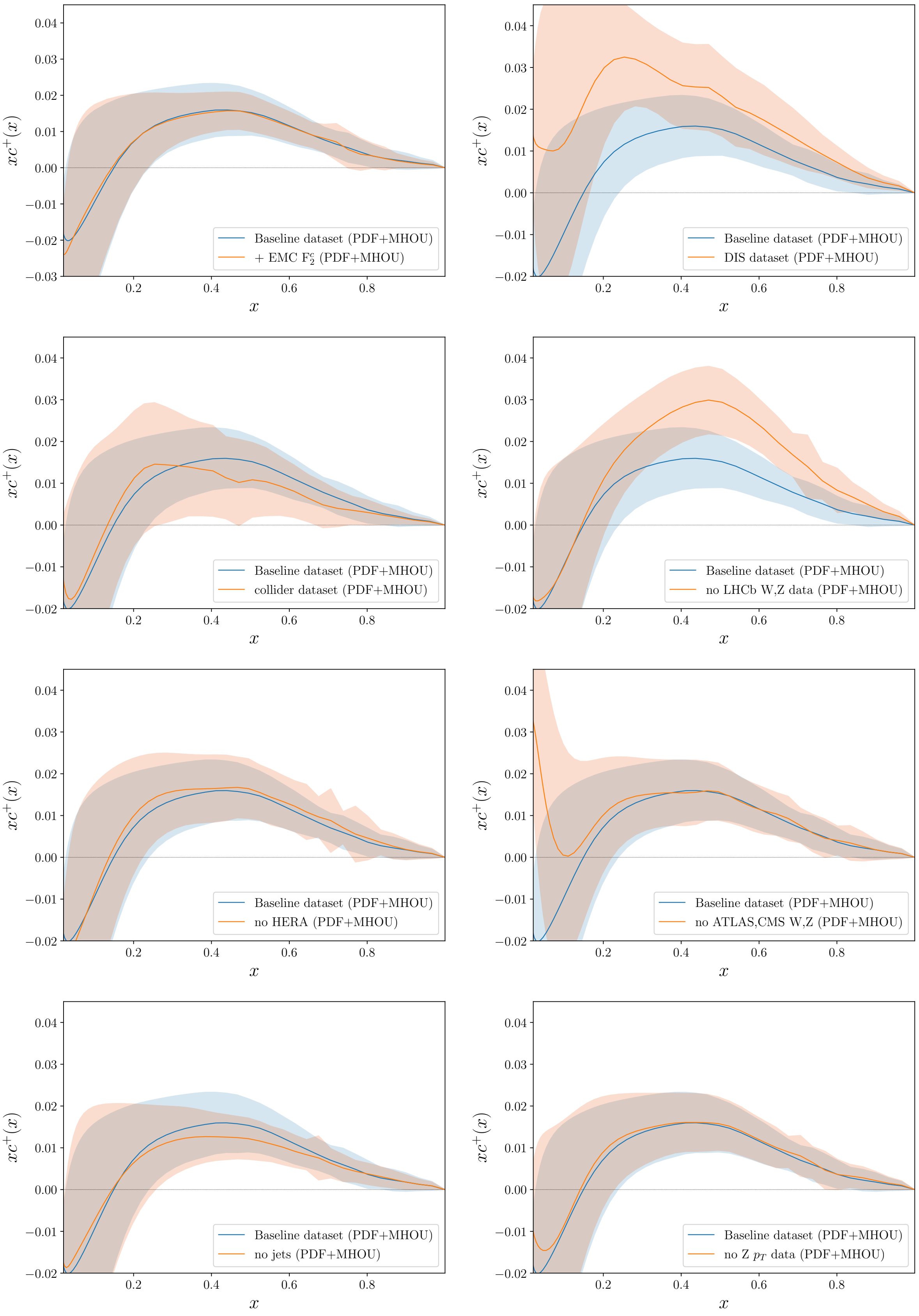

Figure D.1. Same as Fig. C.1 for the intrinsic charm (3FNS) PDF (top four plots), now also including four additional dataset variations: no ATLAS and CMS W, Z production data (third row left), no jet data (third row right), no Z p_T measurements (bottom row left), no HERA DIS data (bottom row right). The error band indicates the PDF uncertainties combined in quadrature with the MHOUs.

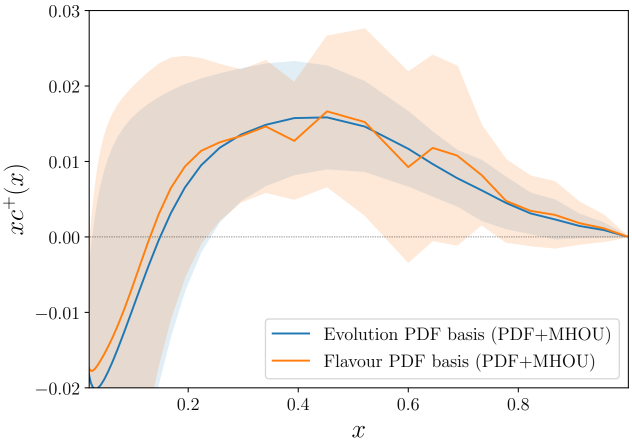

Figure D.2. Same as Fig. C.2 for the intrinsic (3FNS) charm.

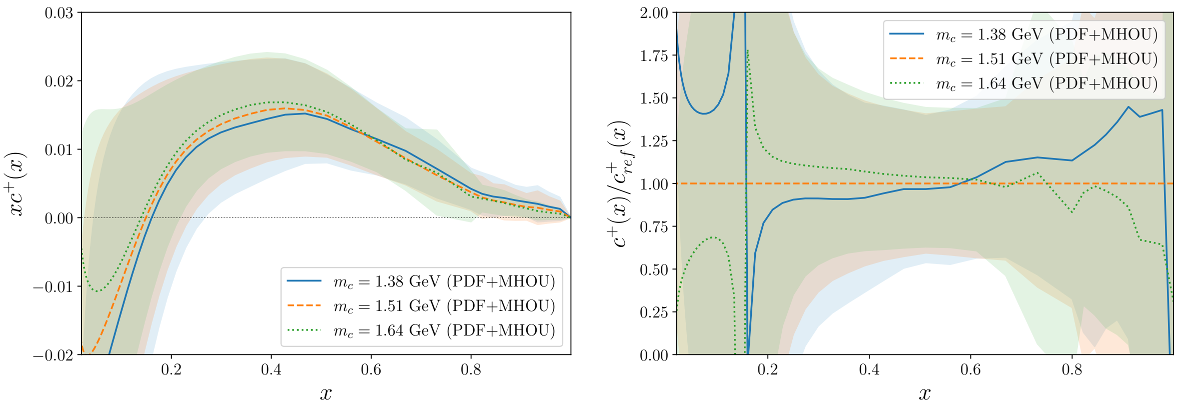

Figure D.3. Same as Fig. C.3, now for the intrinsic (3FNS) charm PDF. Note that the intrinsic charm PDF is scale independent.

Scheme	Q	Charm PDF	m_c	$[c]$ (%)
3FNS	–	default	1.51 GeV	$0.62 \pm 0.28_{\rm pdf} \pm 0.54_{\rm mhou}$
3FNS	–	default	1.38 GeV	$0.47 \pm 0.27_{\rm pdf} \pm 0.62_{\rm mhou}$
3FNS	–	default	1.64 GeV	$0.77 \pm 0.28_{\rm pdf} \pm 0.48_{\rm mhou}$
4FNS	1.65 GeV	default	1.51 GeV	$0.87 \pm 0.23_{\rm pdf}$
4FNS	1.65 GeV	default	1.38 GeV	$0.94 \pm 0.22_{\rm pdf}$
4FNS	1.65 GeV	default	1.64 GeV	$0.84 \pm 0.24_{\rm pdf}$
4FNS	1.65 GeV	perturbative	1.51 GeV	$0.346 \pm 0.005_{\rm pdf} \pm 0.44_{\rm mhou}$
4FNS	1.65 GeV	perturbative	1.38 GeV	$0.536 \pm 0.006_{\rm pdf} \pm 0.49_{\rm mhou}$
4FNS	1.65 GeV	perturbative	1.64 GeV	$0.172 \pm 0.003_{\rm pdf} \pm 0.41_{\rm mhou}$

Table E.1. The charm momentum fraction, Eq. (E.1). We show results both in the 3FNS and the 4FNS (at $Q = 1.65$ GeV) for our default charm, and also in the 4FNS for perturbative charm. We provide results for three different values of the charm mass m_c and indicate separately the PDF and the MHO uncertainties.

E The charm momentum fraction

The fraction of the proton momentum carried by charm quarks is given by

$$[c] = \int_0^1 dx\, xc^+(x, Q^2)\,. \tag{E.1}$$

Model predictions, as mentioned, are typically provided up to an overall normalization, which in turn determines the charm momentum fraction. Consequently, the momentum fraction is often cited as a characteristic parameter of intrinsic charm. It should however be borne in mind that, even in the absence of intrinsic charm, this charm momentum fraction is nonzero due to the perturbative contribution.

In Table E.1 we indicate the values of the charm momentum fraction in the 3FNS for our default charm determination and in the 4FNS (at $Q = 1.65$ GeV) both for the default result and for perturbative charm PDF (see SI Sect. B). We provide results for three different values of the charm mass m_c and indicate separately the PDF and the MHO uncertainties. The 3FNS result is scale-independent, it corresponds to the momentum fraction carried by intrinsic charm, and it vanishes identically by assumption in the perturbative charm case. The 4FNS result corresponds to the scale-dependent momentum fraction that combines the intrinsic and perturbative contribution, while of course it contains only the perturbative contribution in the case of perturbative charm. As discussed in SI Sect. B, the large uncertainty associated to higher order corrections to the matching conditions affects the 3FNS result (intrinsic charm) in the default case, in which the charm PDF is determined from data in the 4FNS scheme, while it affects the 4FNS result for perturbative charm, that is determined assuming the vanishing of the 3FNS result.

For our default determination, the charm momentum fraction in the 4FNS at low scale differs from zero at the 3σ level. However, it is not possible to tell whether this is of perturbative or intrinsic origin, because, due to the large MHOU in the matching condition, the intrinsic (3FNS) charm momentum fraction is compatible with zero. This large uncertainty is entirely due to the small $x \lesssim 0.2$ region, see see Fig. 1 (right). Accordingly, for perturbative charm the low-scale 4FNS momentum fraction is compatible with zero. Consistently with the results of SI Sect. C, the 4FNS result is essentially independent of the value of the charm mass, but it becomes strongly dependent on it if one assumes perturbative charm.

The 4FNS charm momentum fraction is plotted as a function of scale in Fig. E.1, both in the default case and for perturbative charm, with the 3FNS values and the detail of the low-Q 4FNS results shown in an inset. The dependence on the value of the charm mass is shown in Fig. E.2. The large MHOUs on the 3FNS result, and on the 4FNS result in the case of perturbative charm, are apparent. The stability of the default result upon variation of the value of m_c, and

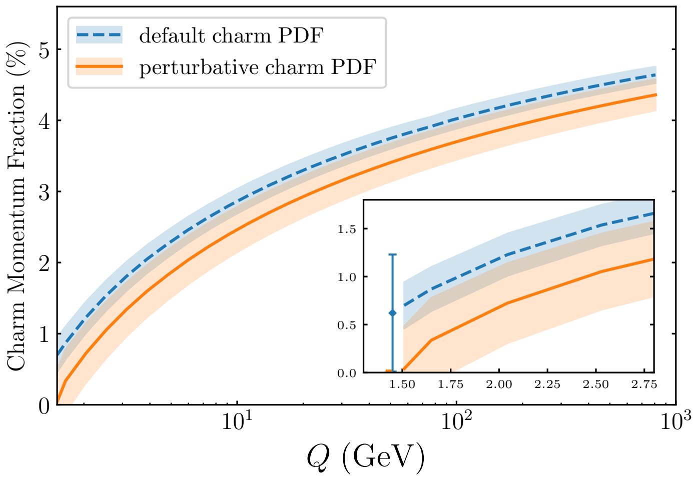

Figure E.1. The 4FNS charm momentum fraction in NNPDF4.0 as a function of scale Q, both for the default and perturbative charm cases, for a charm mass value of $m_c = 1.51$ GeV. The inset zooms on the low-Q region and includes the 3FNS (default) result from Table E.1. Note that the uncertainty includes the MHOU for the 3FNS default and 4FNS perturbative charm cases, while it is the PDF uncertainty for the 4FNS default charm case.

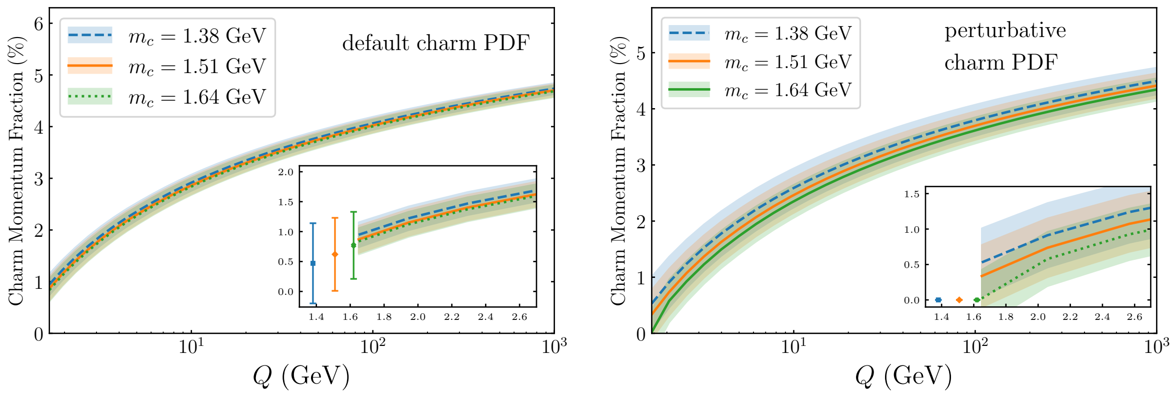

Figure E.2. Same as Fig. E.1 for different values of the charm mass. Note that the 3FNS momentum fraction for perturbative charm vanishes identically by assumption.

the strong dependence of the perturbative charm result on m_c, are also clear. Both the large MHOU uncertainty, and the strong dependence on the value of m_c for perturbative charm are seen to persist up to large scales.

It is interesting to understand in detail the impact of the MHOU on the momentum fraction carried by intrinsic charm. To this purpose, we have computed the truncated momentum integral, i.e. Eq. (E.1) but only integrated down to some lower integration limit $x_{\rm min}$:

$$[c]_{\rm tr}\,(x_{\rm min}) \equiv \int_{x_{\rm min}}^{1} dx\, xc^+(x,Q^2)\,. \tag{E.2}$$

Note than in the 3FNS $xc^+(x)$ does not depend on scale, so this becomes a scale-independent quantity. The result for our default intrinsic charm determination is displayed in Fig. E.3, as a function of of the lower integration limit $x_{\rm min}$. It is clear that for $x_{\rm min} \gtrsim 0.2$ the truncated momentum fraction differs significantly from zero, thereby providing evidence for intrinsic charm with similar statistical significance as the local pull shown in Fig. 2 bottom left. For $x \lesssim 0.2$ this significance is then washed out by the large MHOUs.

Hence, while the total momentum fraction has been traditionally adopted as a measure of intrinsic charm, our analysis shows that, once MHOUs are accounted for, the information provided by the total momentum fraction is limited, at least with current data and theory.

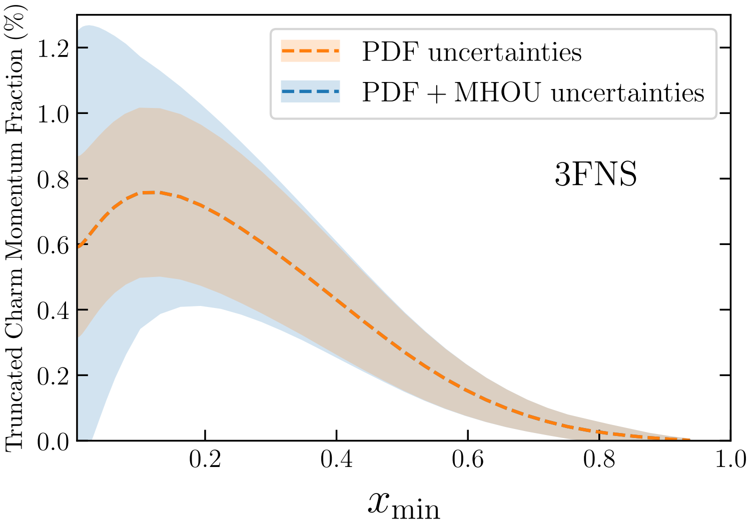

Figure E.3. The value of the truncated charm momentum integral, Eq. (E.2), as a function of the lower integration limit x_{min} for our baseline determination of the 3FNS intrinsic charm PDF. We display separately the PDF and the total (PDF+MHOU) uncertainties.

F Comparison with CT14IC

The possibility of an intrinsic charm component was recently studied in [18], by modifying the CT14 PDF set, with the initial 4FNS charm PDF taken equal to the BHPS model [1] form with the normalization fitted as a free parameter. A 4FNS charm PDF with uncertainties at $Q = 1.3$ GeV was then constructed by taking the BHPS model with best-fit normalization as central value (called the 'BHPS1 model' in [18]); the lower edge of the uncertainty band was taken to coincide with the standard CT14 charm PDF (i.e. the charm PDF determined by perturbative matching from the 3FNS to the 4FNS); the upper edge of the uncertainty band was taken as the BHPS model but with normalization fixed to the upper 90% CL limit (called the 'BHPS2 model' in [18]).

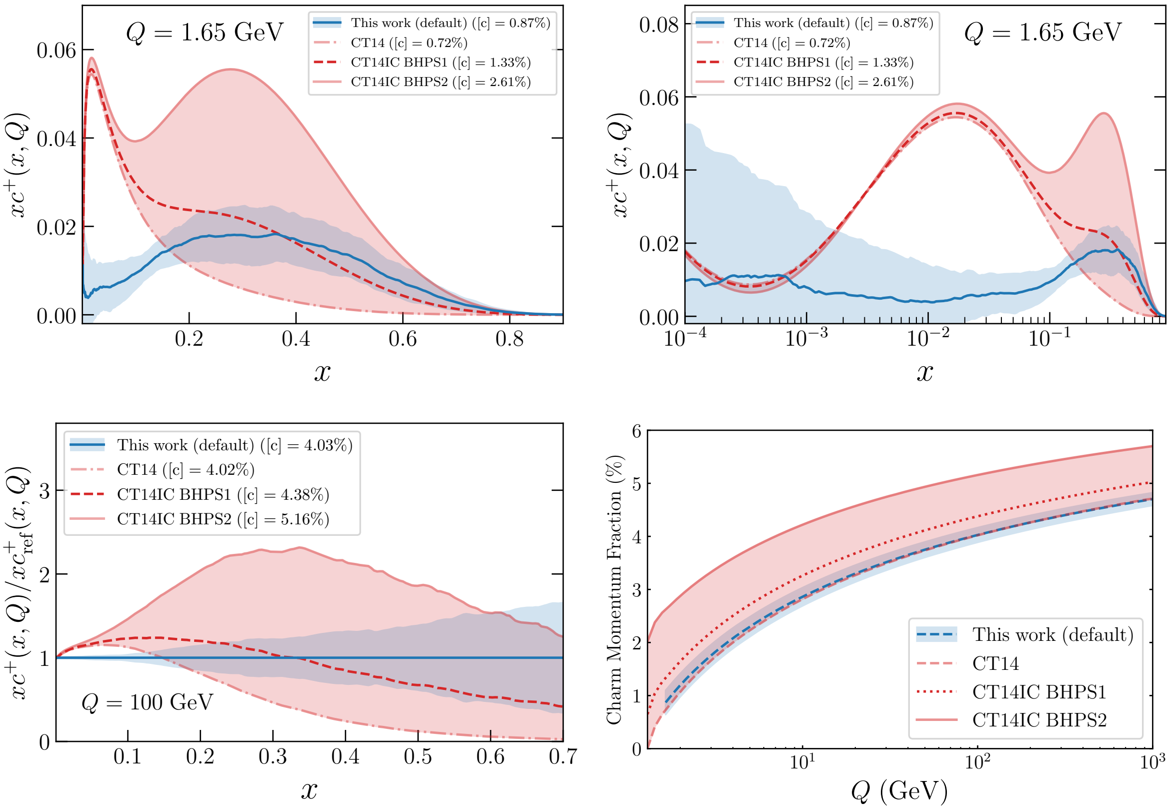

Figure F.1. The 4FNS charm PDF from [18] compared to our result (also in the 4FNS) at $Q = 1.65$ GeV on a linear (top left) and logarithmic (top right) scale in x, and at $Q = 100$ GeV on a linear scale in x and as a ratio to our result (bottom left). The momentum fraction corresponding to either case is also shown as a function of Q (bottom right). Note that for our result the uncertainty band is the 68%CL PDF uncertainty, while for [18] the central curve (labeled CT14IC BHPS1) corresponds to the BHPS model with best-fit normalization, the lower curve (labeled CT14) corresponds to the default CT14 perturbative charm PDF and the upper curve (labeled CT14IC BHPS2) corresponds to the BHPS model with normalization at the upper 90% CL (see text). The value of the momentum fractions are also provided in each case.

The CT14IC charm PDF is compared to our result in Fig. F.1, at $Q = 1.65$ GeV and $Q = 100$ GeV, in the former case on both a logarithmic and linear scale in x and in the latter case on a linear scale only, as a ratio to our default result. Note that the uncertainty band has a different interpretation in the two curves shown: for our result it is the 68% CL PDF uncertainty, while for [18] it corresponds to the model uncertainty estimated as described above. In Fig. F.1 we also quote the charm momentum fraction in each case, at the corresponding scale Q.

As shown in Fig. 1 (right), our result for the charm PDF is in good agreement with the BHPS model at large x. Correspondingly, for $x \gtrsim 0.3$ we find reasonably good agreement between our result and the central curve of [18], which corresponds to a momentum fraction and thus a normalization of the charm PDF not too different from our result (see Table E.1). Both the upper and lower curve from [18] instead do not agree with our result within uncertainties: indeed

the lower edge corresponds to the absence of intrinsic charm (which we exclude) and the upper edge to a momentum fraction which we exclude at more than the 5σ level (see Table E.1).

For intermediate values $3 \cdot 10^{-3} \lesssim x \lesssim 0.3$ our result disagrees with that of [18], while at very small x all results agree, the intrinsic charm being compatible with zero. The disagreement at intermediate x is mostly due to the fact that in [18] charm is assumed to take the BHPS form, which vanishes for $x \lesssim 0.1$, in the 4FNS at the low scale $Q = 1.3$ GeV. Due to perturbative evolution from $Q = 1.3$ GeV to $Q = 1.65$ GeV the charm PDF then develops the large bump that is seen in Fig. F.1, where we instead find that the 4FNS charm PDF is quite small. This difference persists at large scales as seen in Fig. 1 (bottom left).

In terms of momentum fractions, shown in Fig. 1 (bottom right), as already mentioned our result is compatible with the central value of [18] within uncertainties; and also with the lower edge of [18] that corresponds to perturbative charm. The upper edge of the prediction from [18] is instead ruled out at more than 5σ.

G Z+charm production in the forward region

Here we provide full details on our computation of Z+charm production and on the inclusion of the LHCb data for this process in the determination of the charm PDF shown in Fig. 2.

Computational settings. Theoretical predictions for the Z+charm measurements in the forward region by LHCb [6] follow the settings described in [39]. Z+jet events at NLO QCD theory are generated for $\sqrt{s} = 13$ TeV using the Zj package of the POWHEG-BOX [40]. The parton-level events produced by POWHEG are then interfaced to PYTHIA8 [41] with the Monash 2013 tune [65] for showering, hadronization, and simulation of the underlying event and multiple parton interactions. Long-lived hadrons, including charmed hadrons, are assumed stable and not decayed.

Selection criteria on these particle-level events are imposed to match the LHCb acceptance [6]. Z bosons are reconstructed in the dimuon final state by requiring 60 GeV $\leq m_{\mu\mu} \leq$ 120 GeV, and only events where these muons satisfy $p_T^{\mu} \geq 20$ GeV and $2.0 \leq \eta_{\mu} \leq 4.5$ are retained. Stable visible hadrons within the LHCb acceptance of $2.0 \leq \eta \leq 4.5$ are clustered with the anti-k_T algorithm with radius parameter of $R = 0.5$ [66]. Only events with a hardest jet satisfying 20 GeV $\leq p_T^{\rm jet} \leq$ 100 GeV and $2.2 \leq \eta_{\rm jet} \leq 4.2$ are retained. Charm jets are defined as jets containing a charmed hadron, specifically jets satisfying $\Delta R(j, c{-}\text{hadron}) \leq 0.5$ for a charmed hadron with $p_T(c{-}\text{hadron}) \geq 5$ GeV. Jets and muons are required to be separated in rapidity and azimuthal angle, so we require $\Delta R(j, \mu) \geq 0.5$. The resulting events are then binned in the Z bosom rapidity $y_Z = y_{\mu\mu}$.

The physical observable measured by LHCb is the ratio of the fraction of Z+jet events with and without a charm tag,

$$\mathcal{R}_j^c \equiv \frac{\sigma(pp \to Z + \text{charm jet})}{\sigma(pp \to Z + \text{jet})} = \frac{N(c{-}\text{tag})}{N(\text{jets})}\,. \tag{G.1}$$

Here $N(c{-}\text{tag})$ and $N(\text{jets})$ are, respectively, the number of charm-tagged and un-tagged jets, for a Z boson rapidity interval that satisfies the selection and acceptance criteria. The denominator of Eq. (G.1) includes all jets, even those containing heavy hadrons. The charm tagging efficiency is already accounted for at the level of the experimental measurement, so it is not required in the theory simulations.

Predictions for Eq. (G.1) are produced using our default PDF determination (NNPDF4.0 NNLO), as well as the corresponding PDF set with perturbative charm (see SI Sect. B). We have explicitly checked that our results are essentially independent of the value of the charm mass. We have evaluated MHOUs and PDF uncertainties using the output of the POWHEG+PYTHIA8 calculations. We have checked that MHOUs, evaluated with the standard seven-point prescription, essentially cancel in the ratio Eq. (G.1). Note that this is not the case for PDF uncertainties, because the dominant partonic subchannels in the numerator and denominator are not the same.

$\chi^2/N_{\rm dat}$	default charm		perturbative charm	
	$\rho_{\rm sys} = 0$	$\rho_{\rm sys} = 1$	$\rho_{\rm sys} = 0$	$\rho_{\rm sys} = 1$
Prior	1.85	3.33	3.54	3.85
Reweighted	1.81	3.14	–	–

Table G.1. The values of $\chi^2/N_{\rm dat}$ for the LHCb Z+charm data before (prior) and after (reweighted) their inclusion in the PDF fit. Results are given for two experimental correlation models, denoted as $\rho_{\rm sys} = 0$ and $\rho_{\rm sys} = 1$. We also report values before inclusion for the perturbative charm PDFs.

Inclusion of the LHCb data. We first compare the quality of the description of the LHCb data before their inclusion. In Table G.1 we show the values of $\chi^2/N_{\rm dat}$ for the LHCb Z+charm data both with default and perturbative charm. Since the experimental covariance matrix is

not available for the LHCb data we determine the χ^2 values assuming two limiting scenarios for the correlation of experimental systematic uncertainties. Namely, we either add in quadrature statistical and systematic errors ($\rho_{\rm sys} = 0$), or alternatively we assume that the total systematic uncertainty is fully correlated between y_Z bins ($\rho_{\rm sys} = 1$). Fit quality is always significantly better in our default intrinsic charm scenario than with perturbative charm. As is clear from Fig. 2 (top left), the somewhat poor fit quality is mostly due to the first rapidity bin, which is essentially uncorrelated to the amount of intrinsic charm (see Fig. 2, top right).

The LHCb Z+charm data are then included in the PDF determination through Bayesian reweighting [67, 68]. The $\chi^2/N_{\rm dat}$ values obtained using the PDFs found after their inclusion are given in Table G.1. They are computed by combining the PDF and experimental covariance matrix so both sources of uncertainty are included — as mentioned above, MHOUs are negligible. The fit quality is seen to improve only mildly, and the effective number of replicas [67, 68] after reweighting is only moderately reduced, from the prior $N_{\rm rep} = 100$ to $N_{\rm eff} = 92$ or $N_{\rm eff} = 84$ in the $\rho_{\rm sys} = 0$ and $\rho_{\rm sys} = 1$ scenarios respectively. This demonstrates that the inclusion of the LHCb Z+charm measurements affects the PDFs only weakly. This agrees with the results shown in Figs. 2 (center) in the main manuscript, where it is seen that the inclusion of the LHCb data has essentially no impact on the shape of the charm PDF, but it moderately reduces its uncertainty in the region of the valence peak.

H Parton luminosities

The impact of intrinsic charm on hadron collider observables can be assessed by studying parton luminosities. Indeed, the cross-section for hadronic processes at leading order is typically proportional to an individual parton luminosity or linear combination of parton luminosities. Comparing parton luminosities determined using our default PDF set to those obtained imposing perturbative charm (see SI Sect. B) provides a qualitative estimate of the measurable impact of intrinsic charm. Of course this is then modified by higher-order perturbative corrections, which generally depend on more partonic subchannels and thus on more luminosities. In this section we illustrate this by considering the parton luminosities that are relevant for the computation of the Z+charm process in the LHCb kinematics, see SI Sect. G.

The parton luminosity without any restriction on the rapidity y_X of the final state is

$$\mathcal{L}_{ab}(m_X) = \frac{1}{s}\int_{\tau}^{1}\frac{dx}{x} f_a\left(x, m_X^2\right) f_b\left(\tau/x, m_X^2\right) \, , \qquad \tau = \frac{m_X^2}{s} \, , \tag{H.1}$$

where a, b label the species of incoming partons, $\sqrt{s}$ is the center-of-mass energy of the hadronic collision, and m_X is the final state invariant mass. For the more realistic situation where the final state rapidity is restricted, $y_{\rm min} \leq y_X \leq y_{\rm max}$, Eq. (H.1) is modified as

$$\mathcal{L}_{ab}(m_X) = \frac{1}{s}\int_{\tau}^{1}\frac{dx}{x} f_a\left(x, m_X^2\right) f_b\left(\tau/x, m_X^2\right) \theta\left(y_X - y_{\rm min}\right) \theta\left(y_{\rm max} - y_X\right) \, , \tag{H.2}$$

where $y_X = \left(\ln x^2/\tau\right)/2$.

We consider in particular the quark-gluon and the charm-gluon luminosities, defined as

$$\mathcal{L}_{qg}(m_X) \equiv \sum_{i=1}^{n_f}\left(\mathcal{L}_{q_i g}(m_X) + \mathcal{L}_{\bar{q}_i g}(m_X)\right) \, , \quad \mathcal{L}_{cg}(m_X) \equiv \left(\mathcal{L}_{cg}(m_X) + \mathcal{L}_{\bar{c}g}(m_X)\right) \, , \tag{H.3}$$

where n_f is the number of active quark flavors for a given value of $Q = m_X$ with a maximum value of $n_f = 5$. These are the combinations that provide the leading contributions respectively to the numerator ($\mathcal{L}_{cg}$) and the denominator ($\mathcal{L}_{qg}$) of $\mathcal{R}_j^c$ in Eq. (G.1).

The luminosities are displayed in Fig. H.1, in the invariant mass region, 40 GeV $\leq m_X \leq$ 200 GeV which is most relevant for Z+charm production. Results are shown for three different rapidity bins, $-2.5 \leq y_X \leq 2.5$ (central production in ATLAS and CMS), $2.0 \leq y_X \leq 2.75$ (forward production, corresponding to the central bin in LHCb), and $3.5 \leq y_X \leq 4.5$ (highly boosted production, corresponding to the most forward bin in the LHCb selection), as a ratio to our default case.

For central production it is clear that both the quark-gluon and charm-gluon luminosities with our without intrinsic charm are very similar. This means that central Z+charm production in this invariant mass range is insensitive to intrinsic charm. For forward production, corresponding to the central LHCb rapidity bin, $2.0 \leq y_X \leq 2.75$, in the invariant mass region $m_X \simeq 100$ GeV again there is little difference between results with or without intrinsic charm, but as the invariant mass increases the charm-gluon luminosity with intrinsic charm is significantly enhanced. For very forward production, such as the highest rapidity bin of LHCb, $3.5 \leq y_X \leq 4.5$, the charm-gluon luminosity at $m_X \simeq 100$ GeV is enhanced by a factor of about 4 in our default result in comparison to the perturbative charm case, corresponding to a $\simeq 3\sigma$ difference in units of the PDF uncertainty, consistently with the behavior observed for the $\mathcal{R}_j^c$ observable in Fig. 2 (top left) in the most forward rapidity bin. This observation provides a qualitative explanation of the results of SI Sect. G.

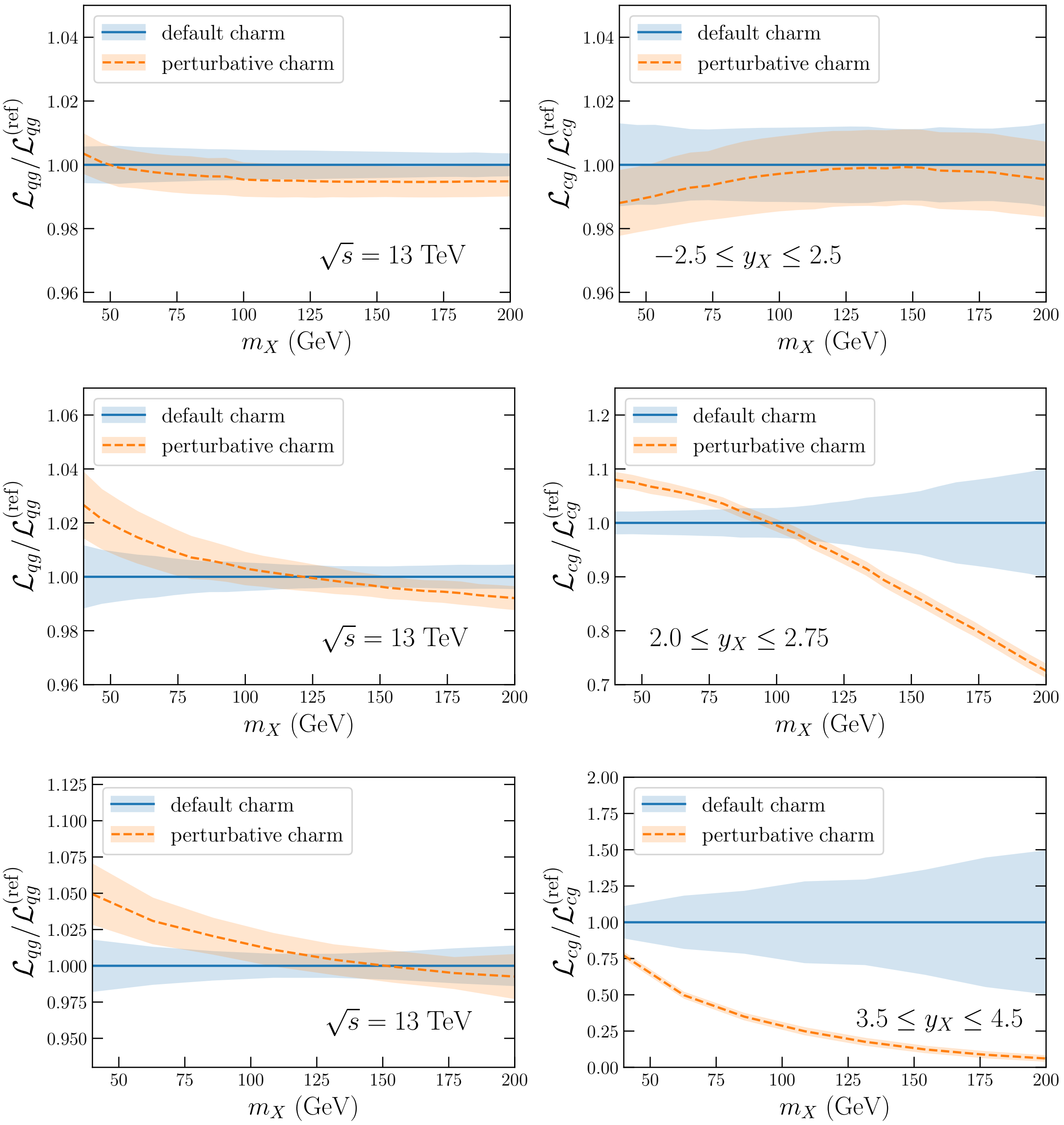

Figure H.1. The quark-gluon (left) and charm-gluon (right) parton luminosities in the m_X region relevant for Z+charm production and three different rapidity bins (see text). Results are shown both for our default charm PDFs and for the variant with perturbative charm.

Supplementary References

[51] **NNPDF** Collaboration, R. D. Ball et al., *An open-source machine learning framework for global analyses of parton distributions*, Eur. Phys. J. C **81** (2021), no. 10 958, [arXiv:2109.02671].

[52] V. Bertone, S. Carrazza, and J. Rojo, *APFEL: A PDF Evolution Library with QED corrections*, Comput.Phys.Commun. **185** (2014) 1647, [arXiv:1310.1394].

[53] A. Candido, F. Hekhorn, and G. Magni, *EKO: Evolution Kernel Operators*, arXiv:2202.02338.

[54] G. P. Salam and J. Rojo, *A Higher Order Perturbative Parton Evolution Toolkit (HOPPET)*, Comput. Phys. Commun. **180** (2009) 120–156, [arXiv:0804.3755].

[55] M. Botje, *QCDNUM: Fast QCD Evolution and Convolution*, Comput.Phys.Commun. **182** (2011) 490–532, [arXiv:1005.1481].

[56] A. Vogt, *Efficient evolution of unpolarized and polarized parton distributions with qcd-pegasus*, Comput. Phys. Commun. **170** (2005) 65–92, [hep-ph/0408244].

[57] **The NNPDF** Collaboration, L. Del Debbio, S. Forte, J. I. Latorre, A. Piccione, and J. Rojo, *Neural network determination of parton distributions: The nonsinglet case*, JHEP **03** (2007) 039, [hep-ph/0701127].

[58] **The NNPDF** Collaboration, R. D. Ball et al., *A determination of parton distributions with faithful uncertainty estimation*, Nucl. Phys. **B809** (2009) 1–63, [arXiv:0808.1231].

[59] **The NNPDF** Collaboration, R. D. Ball et al., *A first unbiased global NLO determination of parton distributions and their uncertainties*, Nucl. Phys. **B838** (2010) 136, [arXiv:1002.4407].

[60] S. Zanoli, *Higher-order matching for heavy quarks in perturbative QCD*, . MSc Thesis, University of Milano, 2020.

[61] M. Dittmar et al., *Working Group I: Parton distributions: Summary report for the HERA LHC Workshop Proceedings*, hep-ph/0511119.

[62] W. Giele et al., *The QCD / SM working group: Summary report*, in 2nd Les Houches Workshop on Physics at TeV Colliders, pp. 275–426, 4, 2002. hep-ph/0204316.

[63] **LHC Higgs Cross Section Working Group** Collaboration, D. de Florian et al., *Handbook of LHC Higgs Cross Sections: 4. Deciphering the Nature of the Higgs Sector*, arXiv:1610.07922.

[64] C. W. Bauer, Z. Ligeti, M. Luke, A. V. Manohar, and M. Trott, *Global analysis of inclusive B decays*, Phys. Rev. D **70** (2004) 094017, [hep-ph/0408002].

[65] P. Skands, S. Carrazza, and J. Rojo, *Tuning PYTHIA 8.1: the Monash 2013 Tune*, European Physical Journal **74** (2014) 3024, [arXiv:1404.5630].

[66] M. Cacciari, G. P. Salam, and G. Soyez, *The Anti-k(t) jet clustering algorithm*, JHEP **0804** (2008) 063, [arXiv:0802.1189].

[67] **The NNPDF** Collaboration, R. D. Ball et al., *Reweighting NNPDFs: the W lepton asymmetry*, Nucl. Phys. **B849** (2011) 112–143, [arXiv:1012.0836].

[68] R. D. Ball, V. Bertone, F. Cerutti, L. Del Debbio, S. Forte, et al., *Reweighting and Unweighting of Parton Distributions and the LHC W lepton asymmetry data*, Nucl.Phys. **B855** (2012) 608–638, [arXiv:1108.1758].
